# Simultaneous determination of trace rare-earth elements in simulated water samples using ICP-OES with TODGA extraction/back-extraction

**DOI:** 10.1371/journal.pone.0185302

**Published:** 2017-09-25

**Authors:** FuKai Li, AiJun Gong, LiNa Qiu, WeiWei Zhang, JingRui Li, Yu Liu, YuNing Liu, HuiTing Yuan

**Affiliations:** School of Chemistry and Biological Engineering, University of Science and Technology Beijing, Beijing, P R, China; University of Edinburgh, UNITED KINGDOM

## Abstract

The determination of trace rare-earth elements (REEs) can be used for the assessment of environmental pollution, and is of great significance to the study of toxicity and toxicology in animals and plants. N, N, N′, N′-tetraoctyl diglycolamide (TODGA) is an environmental friendly extractant that is highly selective to REEs. In this study, an analytical method was developed for the simultaneous determination of 16 trace REEs in simulated water samples by inductively coupled plasma optical emission spectroscopy (ICP-OES). With this method, TODGA was used as the extractant to perform the liquid-liquid extraction (LLE) sample pretreatment procedure. All 16 REEs were extracted from a 3 M nitric acid medium into an organic phase by a 0.025 M TODGA petroleum ether solution. A 0.03 M ethylenediaminetetraacetic acid disodium salt (EDTA) solution was used for back-extraction to strip the REEs from the organic phase into the aqueous phase. The aqueous phase was concentrated using a vacuum rotary evaporator and the concentration of the 16 REEs was detected by ICP-OES. Under the optimum experimental conditions, the limits of detection (3σ, n = 7) for the REEs ranged from 0.0405 ng mL^-1^ (Nd) to 0.5038 ng mL^-1^ (Ho). The relative standard deviations (c = 100 ng mL^-1^, n = 7) were from 0.5% (Eu) to 4.0% (Tm) with a linear range of 4–1000 ng mL^-1^ (R^2^ > 0.999). The recoveries of 16 REEs ranged from 95% to 106%. The LLE-ICP-OES method established in this study has the advantages of simple operation, low detection limits, fast analysis speed and the ability to simultaneously determine 16 REEs, thereby acting as a viable alternative for the simultaneous detection of trace amounts of REEs in water samples.

## Introduction

Rare-earth elements (REEs), according to the International Union of Pure and Applied Chemistry, are defined as including the lanthanides with atomic numbers ranging from 57 (La) to 71 (Lu), and two additional elements, Sc (21) and Y (39) [1]. As a result of their special electronic structural characteristics, REEs have found usage in a variety of electronics, superconductor, microelement fertilizer, ceramic and gasoline-cracking catalyst applications [2–4]. However, with the vast amounts of REEs being used, significant quantities are leaking into the environment, which can then enter the human body via the food chain. It has been reported that lanthanides may impair the stability of DNA and then cause DNA damage, have an effect on the crystallization of urinary stones, induce changes in bone tissue and even cause genetic toxicity in bone marrow cells [5–7]. Therefore, the simultaneous determination of REEs in environmental water samples does not only provide support to the assessment of environmental pollution, but also helps to control the toxicity.

Several analytical methodologies have been applied to the determination of REEs in practical samples, such as neutron activation analysis (NAA) [8–10], X-ray fluorescence (XRF) spectrometry [11–13], inductively coupled plasma mass spectrometry (ICP-MS) [14–17] and inductively coupled plasma optical emission spectrometry (ICP-OES) [18–22]. However, NAA not only requires a nuclear reactor, but also a complicated disposal process, so it is not suitable for common sample detection. The poor sensitivity of XRF also restricts its application in REE determination.

Therefore, ICP-MS and ICP-OES are the most favorable choices for the simultaneous determination of REEs in practical samples. However, ICP-MS can be hampered by isobaric interferences, such as those from barium oxides on Nd, Sm and Eu measurements, and from lighter REE oxides (e.g., CeO and NdO) on heavier REEs (e.g., Gd, Tb and Dy). In addition, its high cost limits its extensive application [23–25]. ICP-OES is used for the determination of REEs because of its inherent capability for rapid simultaneous multi-element detection over a wide range of concentrations [25]. Zereen et al. [17] proposed a 4-(2-thiazolylazo) resorcinol-immobilized Chromosorb 106 for the preconcentration of trace REEs in seawater and estuarine water samples, before detection by ICP-MS. At pH 5.0, the detection limit of the method varied between 0.06 pg mL^-1^ (Pr) and 0.031 pg mL^-1^ (Ce) for the preconcentration of 5.0 mL blank solutions, and the recoveries for the spiked samples ranged between 93 and 105%. Li et al. [26] used co-precipitation with Fe(OH)_3_ to remove the matrix effects from high-salt groundwater and then obtained an accurate determination of sub-ng L^-1^ levels of REEs by ICP-MS. The limits of detection (LODs) of the method ranged from 0.05 pg mL^-1^ for Lu to 0.6 pg mL^-1^ for Nd, with REE recoveries lying in the range of 90 to 125%. Gabriel et al. [25] synthesized calcium alginate beads for Ce, La and Nd preconcentration from groundwater prior to ICP-OES determination and compared the results with a reference method based on ICP-MS. The results showed that the limits of quantification (LOQs) of the method ranged from 6.3 ng mL^−1^ (La) to 15 ng mL^−1^ (Ce), with the recoveries for the spiked samples ranging from 93 to 105%. Seyed et al. [27] used ion-pair microparticles for the preconcentration of 14 REEs and eluted with 2 M HNO_3_, which was directly introduced to ICP-OES for determination. Consequently, LODs were obtained in the range of 0.02–1.00 ng mL^-1^. Spiked concentrations of 10, 20 or 50 ng mL^-1^ were recovered at 90–110%.

As the concentration of REEs in practical samples is typically lower than the detection limit of the instrument, it is difficult or even impossible to directly determine the effects of complicated matrices and the low concentrations of target analytes. Therefore, it is necessary to reduce the detection limit by increasing the preconcentration factor. In practical sample detection, different methods of separation and preconcentration prior to determination by the apparatus have been reported, including ion-exchange resins [28], solid phase extraction [16,18,20–22,29] and cloud point extraction [24,30]. However, these techniques still present some limitations and inconveniences for the analysis, as they are time consuming and tedious. Meanwhile, the concentration of the coexisting ions will significantly increase with increasing preconcentration factor, so that the matrix effects will influence the detection of REEs.

This study presents a new method to further reduce the detection limit. Firstly, the sample was concentrated and dehydrated to obtain a concentrated solution in which the REEs and coexisting ions were enriched simultaneously. In order to remove the coexisting ions, N, N, N′, N′-tetraoctyl diglycolamide (TODGA, petroleum ether as solvent) was used to extract the REEs from the concentrated solution, since TODGA has a strong selective extraction capacity for REEs under certain conditions. Via back-extraction of the organic phase, the REEs entered the aqueous phase. In this case, the interference of coexisting ions was removed, so that the detection limit of the REEs was greatly reduced. Furthermore, this method has the advantages of rapidity, simplicity and sensitivity. Based on this idea, we synthesized a new type of highly efficient and environmentally friendly extractant, TODGA [31], and established a liquid-liquid extraction method that can preconcentrate 16 REEs from high-content matrix elements effectively and determine them by ICP-OES simultaneously. Compared with the obtained results by the method based on ICP-MS determinations, satisfactory results were obtained, indicating that this new method could be an alternative for the simultaneous determination of REEs.

## Materials and methods

### Apparatus

An ICP-OES system (715-ES, Varian Medical Systems, USA) was used for the REE determination. The optimum operating conditions for ICP-OES are summarized in Table 1. Various parameters, such as RF power, plasma gas flow, auxiliary gas flow and nebulizer pressure, were optimized. The analytes were injected through a peristaltic pump. The reference method for 16 REEs in tap water samples was directed analyzed by ICP-MS (NexION 300Q, PE, USA).

**Table 1:** Operating parameters for REE determination using ICP-OES and ICP-MS.

Instrument settings	ICP-OES	ICP-MS
Rf power	1000 W	1100 W
Plasma gas (Ar) flow rate	15 L min^-1^	15 L min^-1^
Auxiliary gas (Ar) flow rate	1.5 L min^-1^	1.2 L min^-1^
Nebulizer gas flow rate	0.6 L min^-1^	0.8 L min^-1^
Nebulizer	Concentric	Concentric
Dwell time	**-**	50 ms
Pump speed	15 rpm	**-**
Nebulizer pressure	200KPa	**-**
Repetition views	2	3
Analytical wavelength (nm)	Ce 418.659, Dy 353.170,	**-**
	Er 337.275, Eu 381.967,	
	Gd 335.047, Ho 345.600	
	La 408.672, Lu 261.542,	
	Nd 401.225, Pr 417.939,	
	Sm 359.259, Tb 350.917,	
	Tm 313.126, Yb 328.937,	
	Y 324.228, Sc 361.383	

### Reagents and standard solutions

TODGA was synthesized in our laboratory by dissolving 15 g of diglycolic in 60 mL of thionyl chloride with a water bath heated 60°C. After 2 h, the excess thionyl chloride was removed under reduced pressure. The product was collected and a 100 mL mixed solution of triethylamine, to which di-n-octylamine was added dropwise with a dropping funnel and then reacted for 2 h. The final reaction solution was washed with acid, base and water, respectively, and then dried to obtain the product. TODGA was characterized by elemental analysis, NMR: (300 MHz, CDCl_3_, Me_4_Si, δ): 4.27 (s, 4H), 3.26 (t, 4H), 3.14 (t, 4H), 1.49 (m, 8H), 1.24 (br, 40H) and 0.88 (t, 12H).

The individual stock standard solutions (1 mg mL^−1^) of 16 REEs (except Pm) were prepared by dissolving appropriate amounts of their SpecPure® oxides (Aladdin Industrial Corporation, Shanghai, China), and diluting to a certain volume with high-purity deionized water. Standard and test solutions were prepared by diluting the stock solution with 5% (v/v) HNO_3_. A solution of 0.025 M TODGA was prepared by dissolving 1.45 g of TODGA in 100 mL of petroleum ether. Petroleum ether (A.R., Sinopharm Chemical Reagent Co., Ltd, China) was used as a disperser solvent. The stock solutions of interfering elements (K^+^, Na^+^, Ca^2+^, Mg^2+^, Fe^3+^, Zn^2+^, Pb^2+^, Cd^2+^, Co^2+^, Cu^2+^ and Mn^2+^) were prepared by dissolving appropriate amounts of their inorganic salts (KNO_3_, NaCl, CaCl_2_, MgSO_4_, Fe(NO_3_)_3_, ZnCl_2_, Pb(NO_3_)_2_, Cd(NO_3_)_2_, CoCl_2_·6H_2_O, Cu(NO_3_)_2_ and MnSO_4_·H_2_O) (A.R., Sinopharm Chemical Reagent Co., Ltd, China) in 5% (w/v) HCl, which were then dissolved using high-purity deionized water to the required volume. All stock standard solutions were stored in volumetric flasks in a refrigerator and maintained at 4°C. All chemicals used were Specpure or at least of analytical grade.

### Analytical method

A tap water sample was collected from the University of Science and Technology Beijing (USTB) and filtered with a 0.45 μm microporous filter membrane to remove insoluble solids. The filtered water sample (200 mL) was concentrated to 20 mL by a vacuum rotary evaporator. A 20 mL was then solution completely transferred to the 250 mL separating funnel and the nitric acid concentration of the sample solution was adjusted to 3 M. A 20 mL solution of 0.025 M TODGA was added and the resulting mixture was shaken for 2 min at ambient temperature. Then, after the layers separated completely, the aqueous layer was collected and the aqueous phase was extracted twice under the same organic phase conditions. The three times organic phase obtained by extraction was combined and 60 mL of a 0.03 M EDTA solution was added and then shaken for 3 min. After the layers separated, the aqueous layer was collected and the organic phase was back-extracted twice under the same conditions. Three times back-extraction solutions containing REEs were combined and concentrated to 5 mL under reduced pressure and then analyzed using ICP-OES.

The blank solution was prepared using high-purity deionized water without the addition of the target REEs, and complied with the same procedure discussed above.

**Fig 1:**
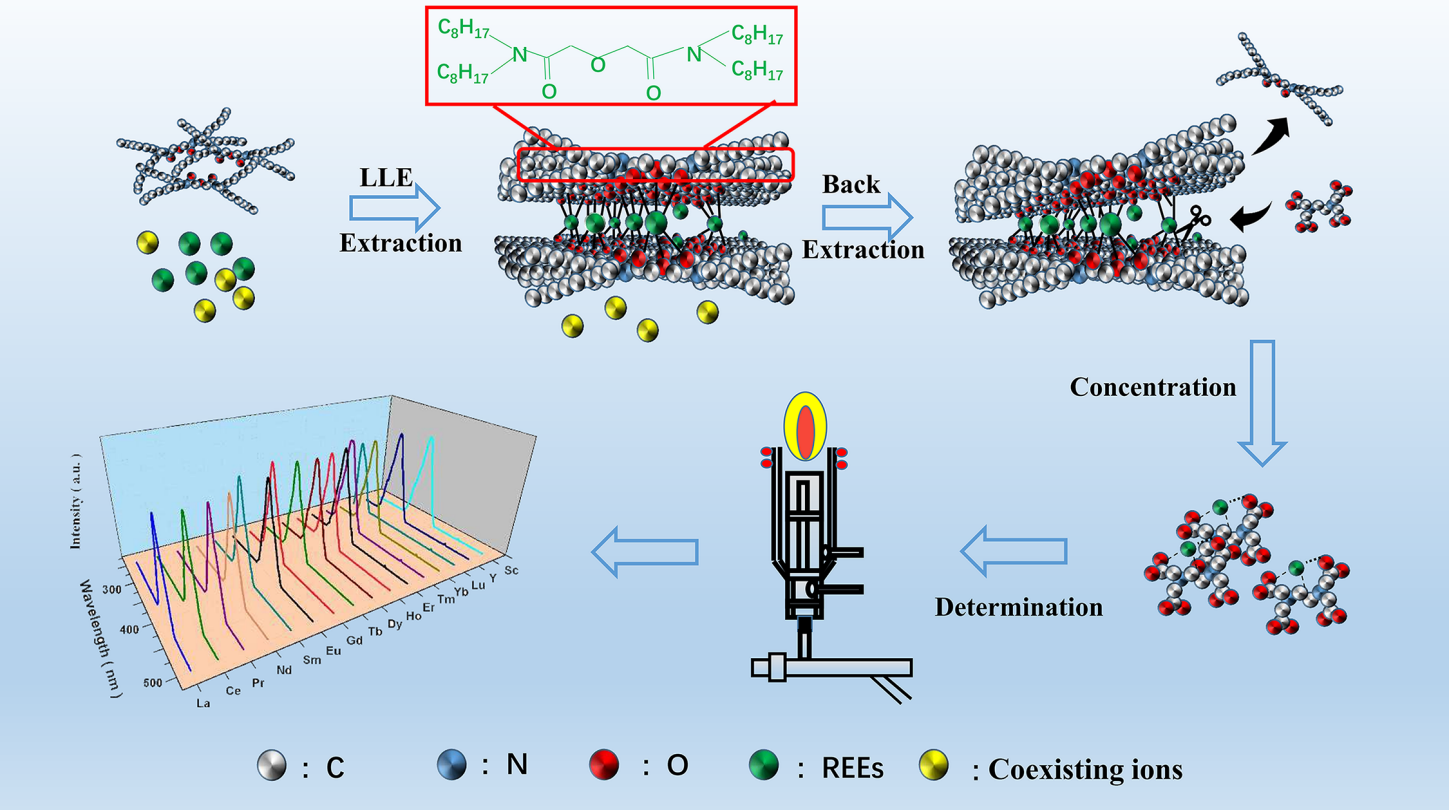
Schematic representation of preconcentration and detection of REEs from a complicated matrix using LLE-ICP-OES.

Before the standard and sample solution introduction to ICP-OES, high-purity deionized water was used for washing to remove the memory of the instrument. A schematic of the LLE-ICP-OES strategy for separation, preconcentration and determination is summarized in Fig 1.

### Batch extraction studies

The concentrations of metal ions in the aqueous phase were measured by ICP-OES (Varian Medical Systems, USA). The concentrations of metal ions in the organic phase were obtained by subtracting the aqueous solution concentration of the analyte from the initial concentration of the analyte. After all extraction processes, the samples were allowed to separate into layers.

The extraction ratio (E%) is evaluated by:

$$E\%=\left(C_{0}-C_{\text{E}}\right)/C_{0}\times 100$$ (1)

where C_0_ and C_E_ are the concentrations of the REEs in the aqueous phase before and after equilibration, respectively.

Back extraction (R%) is evaluated by:

$$R\%=C_{\text{B}}/\left(C_{0}-C_{\text{E}}\right)\times 100$$ (2)

where C_B_ is the back-extraction concentration of the REEs in the aqueous phase.

## Results and discussion

### Effect of HNO_3_ concentration

**Fig 2:**
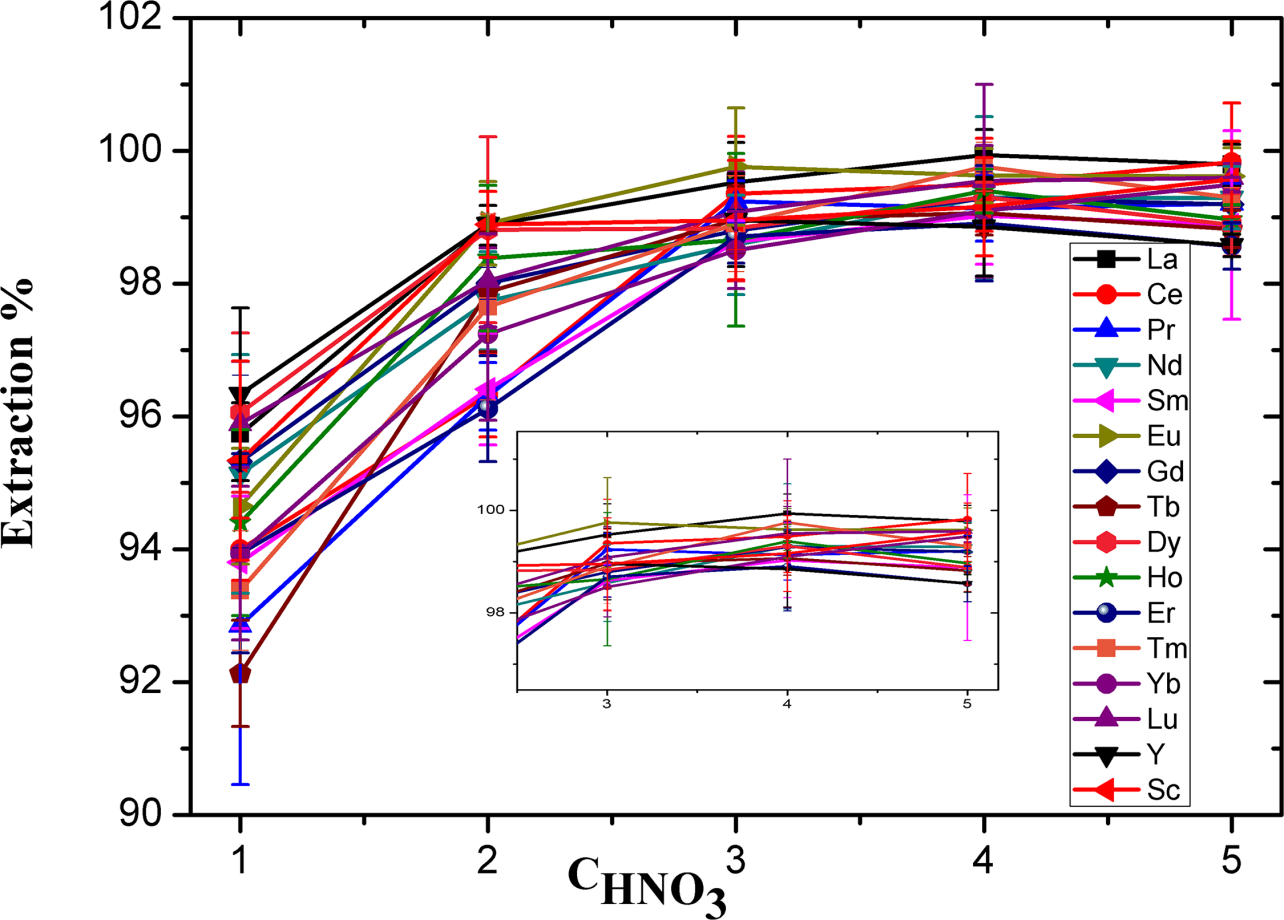
Extraction percentage of REEs as a function of nitric acid concentration in aqueous solution. Concentration of each REE: 200 ng mL^-1^; TODGA: 0.025 M; extraction time: 2 min each time; extraction frequency: three times.

The nitric acid concentration affects the formation of metal chelates and plays an essential role in the extraction of REEs on TODGA. The variation of the extraction ratio (E%) of Ln(III) with the change of the nitric acid concentration is shown in Fig 2. It could be seen that the E% increases gradually with increasing nitric acid concentration and reaches a maximum value at 3 M, where the extraction rate is 98.7–99.9%. Moreover, an increase in the HNO_3_ concentration corresponds to a reduction in the water activity, therefore, the energy required for dehydration of Ln(III) ions decreases so that the TODGA molecules easily form complexes at higher HNO_3_ concentrations [32]. When the concentration of nitric acid increases again, the increasing trend of E% was no longer obvious. Hence, in the following work, 3 M nitric acid was selected in the aqueous phase to ensure complete REE extraction.

### Effect of TODGA concentration

**Fig 3:**
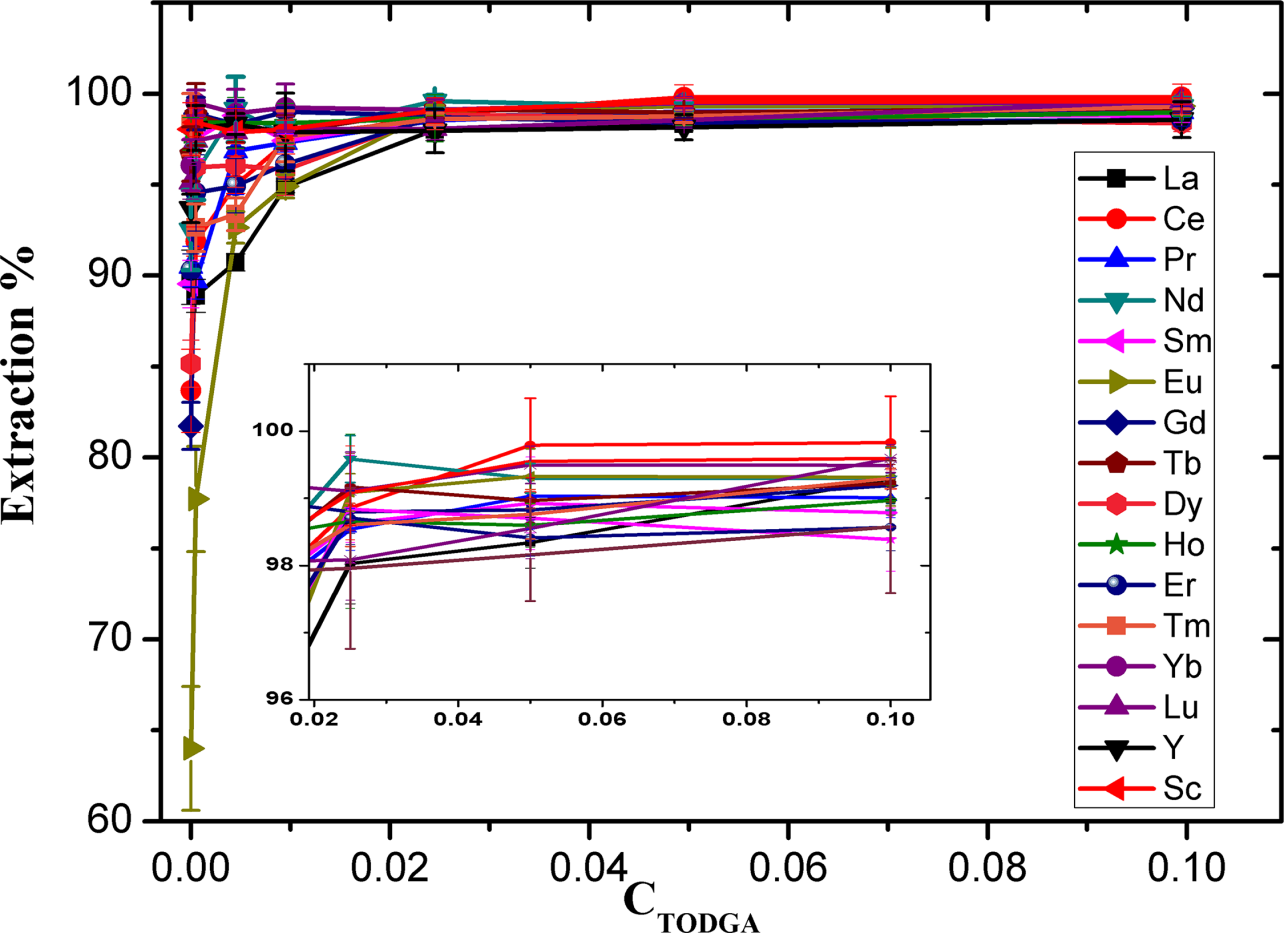
Extraction percentage of REEs as a function of TODGA concentration in the organic phase. Concentration of each REE: 200 ng mL^-1^; concentration of HNO_3_: 3 M; extraction time: 2 min each time; extraction frequency: three times.

The concentration of TODGA was one of the most important factors affecting the extraction efficiency of REEs. The increase of TODGA concentration is beneficial to extraction of REEs, so it is necessary to find the optimum extraction concentration of TODGA. The variation of the E% of Ln(III) as a function of the TODGA concentration was investigated and the results are shown in Fig 3. It can be found that the extraction percentage of the REEs increased with increasing TODGA concentration. When the concentration of TODGA reached 0.025 M, the extraction ratio of the REEs achieved a maximum and thereafter remained constant. Hence, as Fig 3 shows, 0.025 M TODGA was used for further experiments.

### Effect of extraction time

The extraction time could affect the extraction percentage of REEs. Minimizing the extraction time to achieve the best extraction efficiency is of great importance for the fast detection of the method. To achieve the optimized extraction time, the influence of time, varying from 1 to 5 min, on the extraction percentage of the REEs was investigated. The variation of the E% of Ln(III) as a function of the extraction time is shown in S1 Fig. S1 Fig shows that the analyte ion association complex was completed when the extraction time was ~1 min, with the extraction rates of the 16 REEs reaching 93.3–97.8%. This indicates that for the optimized nitric acid and TODGA concentrations, LLE has quick extraction kinetics for REEs. When the extraction time was 2 min, the 16 REEs were basically extracted completely. To achieve the quick and complete extraction of REEs, 2 min was selected as the optimum extraction time.

### Effect of extraction frequency

In order to achieve better extraction efficiency, it was necessary to extract the solution several times. However, an excessive extraction frequency not only consumes the extractant but also time. Therefore, to achieve the optimized extraction frequency, the extraction rates of the REEs under different extraction frequencies were investigated. The results are shown in S2 Fig. As the experimental results show, the extraction efficiencies of some REEs were less than 90% for a single batch contact extraction, with the extraction efficiencies of the REEs increasing with increasing extraction frequencies. The REEs were completely extracted when the extraction frequency was about three times. Hence, as S2 Fig shows, three times was selected for further experiments.

### Effects of coexisting ions

**Fig 4:**
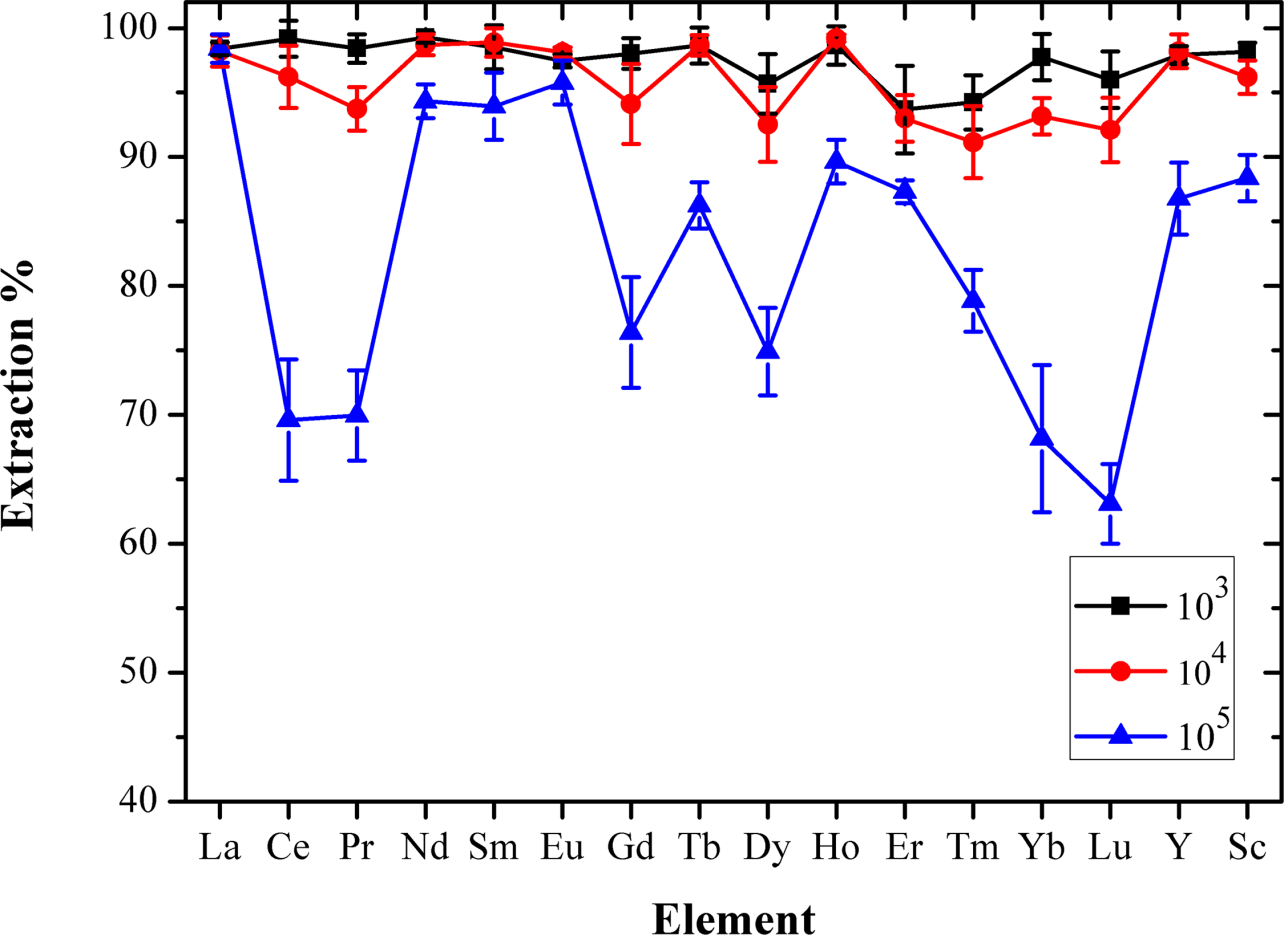
Extraction percentage of REEs as a function of coexisting ion concentration in the aqueous phase. Concentration of each REE: 200 ng mL^-1^; concentration of HNO_3_: 3 M; extraction time: 2min each time; extraction frequency: three times; (10^n^ = C_coexisting ion_/C_REE_, n = 3, 4, 5).

The presence of a highly dissolved matrix in the practical samples not only has a significant matrix effect for target analyte determination, but also affects the extraction efficiency of REEs. Therefore, the common coexisting ions in the actual water samples were added to explore their influence on the extraction of REEs by TODGA, which is used to investigate the anti-interference ability of this method for the detection of trace REEs in the actual sample. Hence, the recovery of REEs in water samples with different concentrations of coexisting ions was studied. For this purpose, coexisting ions, such as K^+^, Na^+^, Ca^2+^, Mg^2+^, Fe^3+^, Zn^2+^, Pb^2+^, Cd^2+^, Co^2+^, Cu^2+^, Mn^2+^, SO_4_^2-^, Cl^-^ and NO_3_^-^, were added to the water samples to explore the effect on the extraction of REEs. The results are shown in Fig 4. When the concentration of each coexisting ion was 2 mg mL^-1^, i.e., C_coexisting ion_/C_REE_ = 10^4^, the extraction rate of the REEs was higher in the range of 91.1–99.2%. Whereas, when the concentration of each coexisting ion was 20 mg mL^-1^, i.e., C_coexisting ion_/C_REE_ = 10^5^, the extraction rate of most REEs decreases obviously, which was within the range of 63.1–98.4%. According to the results, the developed method has excellent selectivity and tolerance for the preconcentration and extraction of REEs in different complex matrices.

### Effect of EDTA concentration on back-extraction

The back-extraction of REEs from the organic to aqueous phase was an essential step, since the organic phase of LLE cannot be directly injected into the ICP-OES for detection. Meanwhile, as the strong extraction of REEs by TODGA, it is difficult to realize the complete back-extraction of all REEs simultaneously. Therefore, selection of the back-extraction solution has an important effect on the stripping of the REEs from TODGA. Several kinds of back-extraction conditions were tested, such as nitric acid, hydrochloric acid and their mixtures at different concentrations. The results show that the back-extraction efficiency was below 80%. Meanwhile, no breakthrough was observed for some heavy REEs, such as Tm and Lu. As a commonly used complexing agent, EDTA has been used to form complexes with multiple ions. Under our experimental conditions, it was found that all the REEs in the organic phase could be simultaneously back-extracted using EDTA.

**Fig 5:**
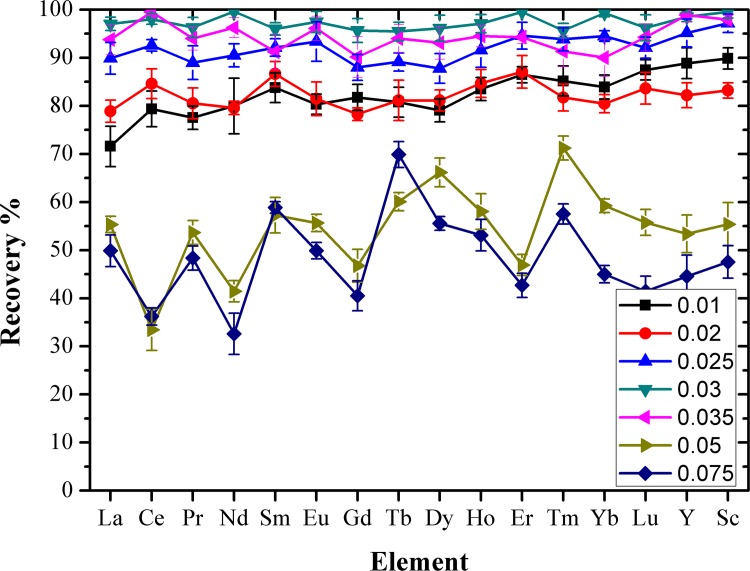
Recovery of REEs as a function of EDTA concentration in the aqueous phase. Concentration of each REE: 200 ng mL^-1^; concentration of HNO_3_: 3 M; extraction time: 2 min; back extraction: 0.03 M EDTA with a stripping time of 3 min.

Fig 5 shows the recovery of REEs with EDTA concentration in the range of 0.010–0.075 M for back extraction of REEs from the organic phase. As could be seen, the recovery of REEs increases with the increase of EDTA concentration, each REE were reached the maximum, respectively. When the concentration of EDTA achieved 0.03 M, whereas, the recovery of REEs decreases as the EDTA concentration continues to increase. This is because after the back-extraction, the EDTA solution needs to be further concentrated at a constant volume, and then detected by ICP-OES. However, a high concentration of EDTA in the solution will precipitate a large amount of solid during the concentration process, which will lead to the loss of REEs and affect their subsequent detection, resulting in lower REE recoveries. It was found that when the concentration of EDTA reached 0.03 M, the REEs in the organic phase had been back extracted completely and there was no solid precipitation after concentrating the back-extraction solution. If the concentration of the EDTA solution continued to increase, there would be a large amount of solid precipitation. This will influence the constant volume of back-extraction solution and the detection of REEs. Accordingly, in order to obtain the optimum recovery rate of REEs, an EDTA concentration of 0.03 M was selected for further experiments.

### Effect of back-extraction time

In order to obtain the optimum back-extraction time during sample processing, the influence of the back-extraction time in the range of 1 to 10 min was evaluated. The variation of the recovery ratio (R%) of 16 REEs as a function of the back-extraction time is shown in S3 Fig. The data presented in S3 Fig show that the back-extraction efficiency distinctly increases with increasing back-extraction time. When the back-extraction time was 1 min, the recoveries of the 16 REEs are in the range of 75.4–89.5%. By continuing to increase the back-extraction time to 2 min, the R% of the REEs are between 90.3 and 96.3%, and when it reaches 3 min, all the 16 REEs were substantially simultaneously stripped into the aqueous phase. There was no significant effect on the stripping when the time continued to increase to 10 min. This tendency indicates that EDTA has fast decomplexation for REEs from TODGA. Thus, in this work, 3 min was chosen for the back-extraction time.

### Effect of back-extraction frequency

Similar to the extraction times, multiple stripping can achieve a better stripping effect for the REEs. Therefore, the effect of back-extraction times was also studied in the range of one to five times. The experimental results presented in S4 Fig show that the recovery of REEs increases with the increase of back-extraction times. As the back-extraction data shows, the recoveries of 16 REEs for single batch contact with the EDTA solution is in the range of 82.1–95.8%. The result shows that the single batch back-extraction cannot fully strip out 16 REEs under the optimum conditions. Therefore, in order to increase the recovery rate of the REEs, it is necessary to continue to increase the back-extraction frequency. When the back-extraction times were up to three times, EDTA basically striped the REEs into the aqueous phase. Therefore, back-extraction for three times was selected as the optimum.

### Analytical performance

Under the optimum experimental conditions, the analytical performance of the method was evaluated to determine its precision and accuracy. The precision of the method was evaluated by the relative standard deviation (RSD) for seven replicate determinations of the 16 REEs at 100 ng mL^−1^. As presented in Table 2, the RSD (n = 7) of 16 REEs at the100 ng mL^−1^ level ranged from 0.5 (Eu) to 4.0% (Tm).

**Table 2:** Analytical characteristics of LLE-ICP-OES method for the determination of the target ions.

Element	Standards added (ng ml^−1^)	Linear equation	Linear range (ng ml^-1^)	Coefficient (R^2^)	LODs^a^ ng ml^-1^ (n = 7)	RSD^b^ (%) (n = 7)	LOQs^c^ ng ml^-1^ (n = 7)	Recovery (%)
La	0,20,200	y = 7.292x+98.603	4–1000	0.9992	0.1378	1.8	0.4134	97.2
Ce	0,20,200	y = 1.297x+56.947	4–1000	0.9994	0.3494	3.6	1.0482	106.8
Pr	0,20,200	y = 1.116x+156.652	4–1000	0.9998	0.2526	2.0	0.7578	98.2
Nd	0,20,200	y = 0.687x+23.334	4–1000	0.9995	0.0405	3.9	0.1215	106
Sm	0,20,200	y = 0.776x+17.881	4–1000	0.9997	0.2275	2.6	0.6825	97.4
Eu	0,20,200	y = 3.791x+10.445	4–1000	0.9999	0.0514	0.5	0.1542	102.4
Gd	0,20,200	y = 1.365x+8.101	4–1000	0.9997	0.4064	3.1	1.2192	100.9
Tb	0,20,200	y = 1.276x+25.798	4–1000	0.9999	0.2409	1.8	0.7227	95.9
Dy	0,20,200	y = 1.867x+16.979	4–1000	0.9999	0.1816	2.1	0.5448	99.4
Ho	0,20,200	y = 1.121x+16.218	4–1000	0.9997	0.5038	3.8	1.5114	102.9
Er	0,20,200	y = 1.442x+13.705	4–1000	0.9996	0.0775	2.4	0.2325	99.1
Tm	0,20,200	y = 3.502x+34.648	4–1000	0.9999	0.2913	4.0	0.8739	97.3
Yb	0,20,200	y = 1.861x+18.328	4–1000	0.9999	0.0738	1.6	0.2214	100.9
Lu	0,20,200	y = 7.302x+2.87	4–1000	0.9999	0.0998	2.1	0.2994	103.9
Y	0,20,200	y = 3.158x+15.992	4–1000	0.9998	0.2187	3.2	0.6561	100.1
Sc	0,20,200	y = 6.694x+24.293	4–1000	0.9996	0.0483	1.9	0.1449	103.5

^a^LODs: Limits of detection.

b: obtained for a concentration of 100 ng mL^−1^.

^c^LOQs: Limits of quantification

The calibration curves of each REE, as an important analytical parameter, were obtained by the calibration solution to ICP-OES. As shown in Table 2, the linear dynamic range of this method was from 4 to 1000 ng mL^-1^ with a correlation coefficient (r^2^) higher than 0.999, which means there was a good linear relationship between the signal intensities of the REEs and their concentrations.

Under the optimum experimental conditions, the LODs of the developed method was obtained based on three times the standard deviations (3σ) for seven replicates determination of the blanks. The experimental results are shown in Table 2. The data presented in Table 2 show that the LODs of the 16 REEs ranged from 0.0405 ng mL^-1^ (Nd) to 0.5038 ng mL^-1^ (Ho). The limits of quantification (LOQs) of the method was based on three times the LODs. Table 2 presents the data of 16 REEs, showing that the LOQs of the method ranged from 0.1215 ng mL^-1^ (Nd) to 1.5114 ng mL^-1^ (Ho).

In order to validate the accuracy of a method, certified reference materials are frequently used. Unfortunately, due to the non-availability of certified reference materials of practical waters and low concentrations of REEs in practical samples, in this study, the standard addition method was selected. A series of tap water samples spiked with various concentrations of REEs (0, 20 and 200 ng mL^−1^) were prepared, and then, the extraction and back-extraction of the samples were carried out under the optimum conditions. The accuracy of this proposed methodology was determined by investigating the standard addition recoveries and the results are summarized in Table 2. The sample recovery rates were 95.9% (Tb) to 106.8% (Ce), which means that each REE could be quantitatively recovered with stable efficiency in the explored concentration range of REEs. Table 3 presents a comparison of the analytical performance data of this method with the published literature. As can be seen, the LODs of this study (0.0405–0.5038 ng mL^-1^) for the 16 REEs (except Pm) were lower than Ref. [25] of 0.02–1 ng mL^-1^ and Ref. [30] of 0.04–0.92 ng mL^-1^. Meanwhile, comparable with the reported in Refs. [18] of 0.01–0.42 ng mL^-1^ and Ref. [22] of 0.13–0.41 ng mL^-1^. Whereas, the REEs determined by this method were more than that of Refs. [18,22,24,25,30]. Therefore, compared with the reported methods, the LLE-ICP-OES method established in this study has the advantages of simple operation, low LODs, selectivity (Fig 4), fast analysis speed and simultaneous determination of 16 REEs, thereby providing an alternative method for the simultaneous detection of trace amounts of REEs in water samples with complex matrices.

**Table 3:** Comparison of the proposed LLE-ICP-OES procedure with other analytical methods.

Preconcentration	Detection method	Element	REEs	LODs ng ml^-1^	Samples	Reference
Amberlite XAD-4	ICP-OES	Pr, Sm, Eu, Gd, Tb, Dy, Ho, Tm, Yb, Lu	10	0.01–0.42	Tap water sea water	[18]
Graphene oxide-TiO_2_ composite	ICP-OES	La, Ce, Eu, Dy, Yb	5	0.13–0.41	Lake water, river water,	[22]
Calcium alginate beads	ICP-OES	La, Ce, Nd,	3	-	Groundwater	[25]
Ion-pair microparticles	ICP-OES	La, Ce, Pr, Nd, Sm, Eu, Gd, Tb, Dy, Ho, Er, Tm, Yb, Lu	14	0.02–1	Well water	[27]
Micelle-mediated extraction		La, Nd, Sm, Gd, Ho, Er, Yb	7	0.04–0.92	Waters	[33]
Liquid-liquid extraction	ICP-OES	La, Ce, Pr, Nd, Sm, Eu, Gd, Tb, Dy, Ho, Er, Tm, Yb, Lu, Y, Sc	16	0.0405–0.5038	Tap water	this study

### Determination of REEs in simulated water samples

Two simulated water samples were prepared by spiking them with two concentrations of REEs (20 and 200 ng mL^−1^) in filtered tap water. Under the optimum experimental conditions, the accuracy of this proposed LLE-ICP-OES methodology was determined by the recovery of the REEs and then compared with the ICP-MS reference method. The results are summarized in Table 4. As can be seen, the recoveries of the REEs spiked in the tap water samples were found to be 95–106%, which indicated the reliability of the LLE-ICP-OES method for the determination of REEs in practical water samples with complex matrices, providing an alternative method for the detection of actual environmental samples.

**Table 4:** Comparison of the recovery of tap water samples spiked with different concentrations of REEs using the proposed LLE-ICP-OES method and the ICP-MS reference method.

REEs	Tap water sample			
	Added (ng ml^−1^)	ICP-MS (mean ± sd, n = 3)	LLE-ICP-OES (mean ± sd, n = 3)	Recovery (%)
La	20	20.4 ± 3.5	19.4 ± 2.1	97.2%
	200	213.1 ± 1.2	190 ± 1	95.4%
Ce	20	22.7 ± 3.8	21.1 ± 0.6	106.8%
	200	207.2 ± 1	198.8 ± 0.5	99.5%
Pr	20	20.2 ± 4.2	19.5 ± 1.2	98.2%
	200	205.1 ± 0.7	198.5 ± 0.3	99.3%
Nd	20	21.1 ± 3.2	21.2 ± 0.8	106%
	200	201.7 ± 0.8	202.5 ± 0.6	101.4%
Sm	20	19.7 ± 3.0	19.1 ± 0.7	97.4%
	200	195.1 ± 0.7	199.7 ± 1.5	99.8%
Eu	20	20.8 ± 3.4	20.1 ± 1.5	102.4%
	200	210.3 ± 1.1	200.7 ± 1.6	100.5%
Gd	20	21 ± 3.6	20.1 ± 2.3	100.9%
	200	205.3 ± 1	210.4 ± 1.3	105.7%
Tb	20	21.3 ± 4.4	19.1 ± 0.4	95.9%
	200	212.2 ± 1.1	196.1 ± 0.6	98.1%
Dy	20	19.3 ± 3.9	19.7 ± 0.6	99.4%
	200	203.5 ± 1.2	200.8 ± 0.7	100.5%
Ho	20	23.1 ± 3.8	20.5 ± 3.1	102.9%
	200	211.2± 1.5	201.2 ± 1.9	100.8%
Er	20	21.5 ± 3.8	19.9 ± 1	99.1%
	200	202.3 ± 1.4	195.3 ± 2.6	97%
Tm	20	20.3 ± 4.0	19.5 ± 0.7	97.3%
	200	201.3 ± 1.7	197.3 ± 1.6	98.5%
Yb	20	20.7 ± 3.5	20.2 ± 0.5	100.9%
	200	206.4 ± 1.3	200.4 ± 0.07	100.2%
Lu	20	21.4 ± 3.3	20.3 ± 1.2	103.9%
	200	210.5 ± 1.5	194.1 ± 2	96.8%
Y	20	20.8 ± 3.7	20 ± 0.5	100.1%
	200	198.4 ± 1.5	202.4 ± 0.2	101.3%
Sc	20	21.2 ± 1.9	20.7 ± 1.4	103.5%
	200	204.3 ± 2.3	198.8 ± 0.5	99.4%

## Conclusions

An analytical method based on environmentally-friendly and efficient TODGA as an extractant with ICP-OES was used to simultaneously detect 16 REEs in simulated water samples. The extraction and back-extraction behavior of the REEs on TODGA was systematically investigated. The LLE-ICP-OES method established in this work has the advantages of simple operation, fast analysis speed, fast extraction and stripping kinetics for REEs. Compared with other methods, this methodology has the ability to simultaneously determine 16 REEs and possesses low detection limits, a wide range of linearity, good anti-interference ability, which provides a good choice for the preconcentration, and separation detection of REEs in actual water samples with complex matrices.

## Supporting information

S1 Fig Extraction percentage of REEs as a function of extraction time in the organic phase. Concentration of each REE: 200 ng mL^-1^; concentration of HNO_3_: 3 M; TODGA: 0.025 M; extraction frequency: three times.
(TIF)

S2 Fig Extraction percentage of REEs as a function of extraction frequency in the organic phase. Concentration of each REE: 200 ng mL^-1^; concentration of HNO_3_: 3 M; TODGA: 0.025 M; extraction time: 2 min each time.
(TIF)

S3 Fig Recovery of REEs as a function of back-extraction time in the aqueous phase. Concentration of each REE: 200 ng mL^-1^; concentration of HNO_3_: 3 M; extraction time: 2 min each time; back extraction: 0.03 M EDTA three times.
(TIF)

S4 Fig Recovery of REEs as a function of back-extraction frequency in the aqueous phase. Concentration of each REE: 200 ng mL^-1^; concentration of HNO_3_: 3 M; extraction time: 2 min each time; back extraction: 0.03 M EDTA with a stripping time of 3 min.
(TIF)

## References

[1] Connelly NG. Nomenclature of inorganic chemistry–IUPAC recommendations 2005. Chemistry International—Newsmagazine for IUPAC. 2007; 27: 25.

[2] Li Y, Yang JL, Jiang Y. Trace rare earth element detection in food and agricultural products based on flow injection walnut shell packed microcolumn preconcentration coupled with inductively coupled plasma mass spectrometry. J Agr Food Chem. 2012; 60: 3033–3041. doi: 10.1021/jf2049646 22390234 10.1021/jf2049646

[3] Sereshti H, Far AR., Samadi S. Optimized ultrasound-assisted emulsification-microextraction followed by ICP-OES for simultaneous determination of lanthanum and cerium in urine and water samples. Anal Lett. 2012; 45: 1426–1439. doi: 10.1080/00032719.2012 10.1016/j.talanta.2012.04.024 22841073

[4] Guo XQ, Tang XT, He M, Chen BB, Nan K, Zhang QY, et al Dual dispersive extraction combined with electrothermal vaporization inductively coupled plasma mass spectrometry for determination of trace REEs in water and sediment samples. RSC Adv. 2014; 4: 19960–19969. doi: 10.1039/C4RA01576B

[5] Aniwashi J, Kaleri HA, Sulaiman Y, Qi-Fa L, Zhuang X. Interactions between rare-earth ions and DNA of Bashibai sheep. Genet Mol Res. 2011; 10: 1075−1083. doi: 10.4238/vol10-2gmr1070 21710458 10.4238/vol10-2gmr1070

[6] Wen HR, Chen RS, in: Collrey P, Bratter P, Bratter VN, Khassanova L, Etienne JC, (Eds.). Paris: Metal Ions in Biology and Medicine, John Libbey Eurotext, 1998; 199.

[7] Cai DJ, Rui YK, Determination of rare earth elements in Camellia oleifera seeds from rare earth elements mining areas in Southern Jiangxi, China by ICP-MS. J Verbrauch Lebensm. 2011; 6: 349–351. doi: 10.1007/s00003-010-0650-7

[8] El-Taher A, Rare-earth elements in Egyptian granite by instrumental neutron activation analysis, Appl Radiat Isotopes. 2007; 65: 458–64. doi: 10.1016/j.apradiso.2006 10.1016/j.apradiso.2006.07.014 17208446

[9] Tao L, Li K, Wang L. State of rare earth elements in different environmental components in mining areas of China. Environ Monit Assess. 2014; 186: 1499–1513. doi: 10.1007/s10661-013-3469-8 24135922 10.1007/s10661-013-3469-8

[10] Kumar K, Saion E, Halimah M, Yap CK, Hamzah MS. Rare earth element (REE) in surface mangrove sediment by instrumental neutron activation analysis, J Radioanal Nucl Chem. 2014; 301: 667–676. doi: 10.1007/s10967-014-3221-z

[11] Sitko R, Zawisza B, Czaja M. Fundamental parameters method for determination of rare earth elements in apatites by wavelength-dispersive X-ray fluorescence spectrometry, J Anal Atom Spectrom. 2005; 20: 741–745. doi: 10.1039/b502994e

[12] De Vito IE, Masi AN, Olsina RA. Determination of trace rare earth elements by X-ray fluorescence spectrometry after preconcentration on a new chelating resin loaded with thorin. Talanta. 1999; 49: 929 doi: 10.1016/S0039-9140(99)00089-2 18967670 10.1016/s0039-9140(99)00089-2

[13] De Vito IE, Olsina RA, Masi AN. Preconcentration and elimination of matrix effects in XRF determinations of rare earth elements by preparing a thin film through chemofiltration. J Anal Atom Spectrom. 2001; 16: 275–278. doi: 10.1039/b008165p

[14] Donard A, Pottin AC, Pointurier F, Pécheyran C. Determination of relative rare earth element distributions in very small quantities of uranium ore concentrates using femtosecond UV laser ablation–SF-ICP-MS coupling. J Anal Atom Spectrom. 2015; 30: 2420–2428. doi: 10.1039/C5JA00309A

[15] Zhu Y, Inagaki K, Haraguchi H, Chiba K. On-line elution of iron hydroxide coprecipitate carrier for determination of REEs in natural water by mix-gas ICP-MS. J Anal Atom Spectrom. 2010; 25: 364–369. doi: 10.1039/B921214K

[16] Su S, Chen B, He M, Hu B, Xiao Z. Determination of trace/ultratrace rare earth elements in environmental samples by ICP-MS after magnetic solid phase extraction with Fe_3_O_4_@ SiO_2_@ polyaniline–graphene oxide composite. Talanta. 2014; 119: 458–466. doi: 10.1016/j.talanta.2013.11.027 24401441 10.1016/j.talanta.2013.11.027

[17] Zereen F, Yilmaz V, Arslan Z. Solid phase extraction of rare earth elements in seawater and estuarine water with 4-(2-thiazolylazo) resorcinol immobilized Chromosorb 106 for determination by inductively coupled plasma mass spectrometry. Microchem J. 2013; 110: 178–184. doi: 10.1016/j.microc.2013.03.012 24000264 10.1016/j.microc.2013.03.012 PMC3758135

[18] Karadaş C, Kara D. Determination of Rare Earth Elements by Solid Phase Extraction Using Chemically Modified Amberlite XAD-4 Resin and Inductively Coupled Plasma-Optical Emission Spectrometry. Water Air Soil Pollt. 2014; 225: 2192 doi: 10.1007/s11270-014-2192-6

[19] Zhao X, Kong X, Jia Q. Combination of on-line preconcentration by di(2-ethylhexyl) phosphoric acid resin in the presence of complexing agent with microwave plasma-atomic emission spectrometry for the determination of rare earths. J Rare Earth. 2010; 28: 79–82. doi: 10.1016/S1002-0721(10)60342-0

[20] Tajabadi F, Yamini Y, Sovizi MR. Carbon-based magnetic nanocomposites in solid phase dispersion for the preconcentration some of lanthanides, followed by their quantitation via ICP-OES. Microchim Acta. 2013; 180: 65–73. doi: 10.1007/s00604-012-0913-3

[21] Berijani S, Ganjali MR, Sereshti H, Tabatabaei SH, Norouzi P. Application of a new modified magnetic nanoparticle as a selective sorbent for preconcentration and extraction of europium in environmental water samples prior to ICP-OES determination. J Iran Chem Soc. 2015; 12: 737–742. doi: 10.1007/s13738-014-0532-5

[22] Zhang Y, Zhong C, Zhang Q, Chen B, He M, Hu B. Graphene oxide–TiO_2_ composite as a novel adsorbent for the preconcentration of heavy metals and rare earth elements in environmental samples followed by on-line inductively coupled plasma optical emission spectrometry detection. RSC Adv. 2015; 5: 5996–6005. doi: 10.1039/C4RA13333A

[23] Dulski P. Interferences of oxide, hydroxide and chloride analyte species in the determination of rare earth elements in geological samples by inductively coupled plasma-mass spectrometry. Fresen J Anal Chem. 1994; 350: 194–203.

[24] Li Y, Hu B. Cloud point extraction with/without chelating agent on-line coupled with inductively coupled plasma optical emission spectrometry for the determination of trace rare earth elements in biological samples. J Hazard Mater. 2010; 174: 534–540. doi: 10.1016/j.jhazmat.2009.09.084 19815341 10.1016/j.jhazmat.2009.09.084

[25] Arantes de Carvalho GG, Kondaveeti S, Petri DF, Fioroto AM, Albuquerque LG, Oliveira PV. Evaluation of calcium alginate beads for Ce, La and Nd preconcentration from groundwater prior to ICP OES analysis. Talanta. 2016; 161: 707–712. doi: 10.1016/j.talanta.2016.09.027 27769469 10.1016/j.talanta.2016.09.027

[26] Li YT, Guo W, Wu ZW, Jin LL, Ke YQ, Guo QH, et al Determination of ultra-trace rare earth elements in high-salt groundwater using aerosol dilution inductively coupled plasma-mass spectrometry (ICP-MS) after iron hydroxide co-precipitation. Microchem J. 2016; 126: 194–199. doi: 10.1016/j.microc.2015.08.009

[27] Yousefi SR, Zolfonoun E, Pourjavid MR, Ahmadi SJ. On-line surfactant-based extraction using ion-pair microparticles combined with ICP-OES for simultaneous preconcentration and determination of rare earth elements in aqueous samples. Anal Methods. 2014; 6: 3694–3699. doi: 10.1039/C4AY00176A

[28] Jia Q, Kong X, Zhou W, Bi L. Flow injection on-line preconcentration with an ion-exchange resin coupled with microwave plasma torch-atomic emission spectrometry for the determination of trace rare earth elements. Microchem J. 2008; 89: 82–87. doi: 10.1016/j.microc.2007.12.005

[29] Pyrzynska K, Kubiak A, Wysocka I. Application of solid phase extraction procedures for rare earth elements determination in environmental samples. Talanta. 2016; 154: 15–22. doi: 10.1016/j.talanta.2016.03.022 27154643 10.1016/j.talanta.2016.03.022

[30] Labrecque C, Larivière D. Quantification of rare earth elements using cloud point extraction with diglycolamide and ICP-MS for environmental analysis. Anal Methods. 2014; 6: 9291–9298. doi: 10.1039/C4AY01911C

[31] Ansari SA, Pathak P, Mohapatra PK, Manchanda VK. Chemistry of diglycolamides: promising extractants for actinide partitioning. Chem Rev. 2012; 112: 1751–1772. doi: 10.1021/cr200002f 22053797 10.1021/cr200002f

[32] Husain M, Ansari SA, Mohapatra PK, Gupta RK, Parmar VS, Manchanda VK. Extraction chromatography of lan-thanides using N, N, N′, N′-tetraoctyl diglycolamide (TODGA) as the stationary phase. Desalination. 2008; 229: 294–301. doi: 10.1016/j.desal.2007.10.016

[33] Hassanien MM, Kenawy IMM, Khalifa ME, Elnagar MM. Mixed micelle-mediated extraction approach for matrix elimination and separation of some rare earth elements. Microchem J. 2016; 127: 125–132. doi: 10.1016/j.microc.2016.02.014

