# Supplementary figures and images for: Simultaneous determination of trace rare-earth elements in simulated water samples using ICP-OES with TODGA extraction/back-extraction
Source: PLoS One. 2017 Sep 25;12(9):e0185302. doi: 10.1371/journal.pone.0185302 (PMC5612762; doi:10.1371/journal.pone.0185302)

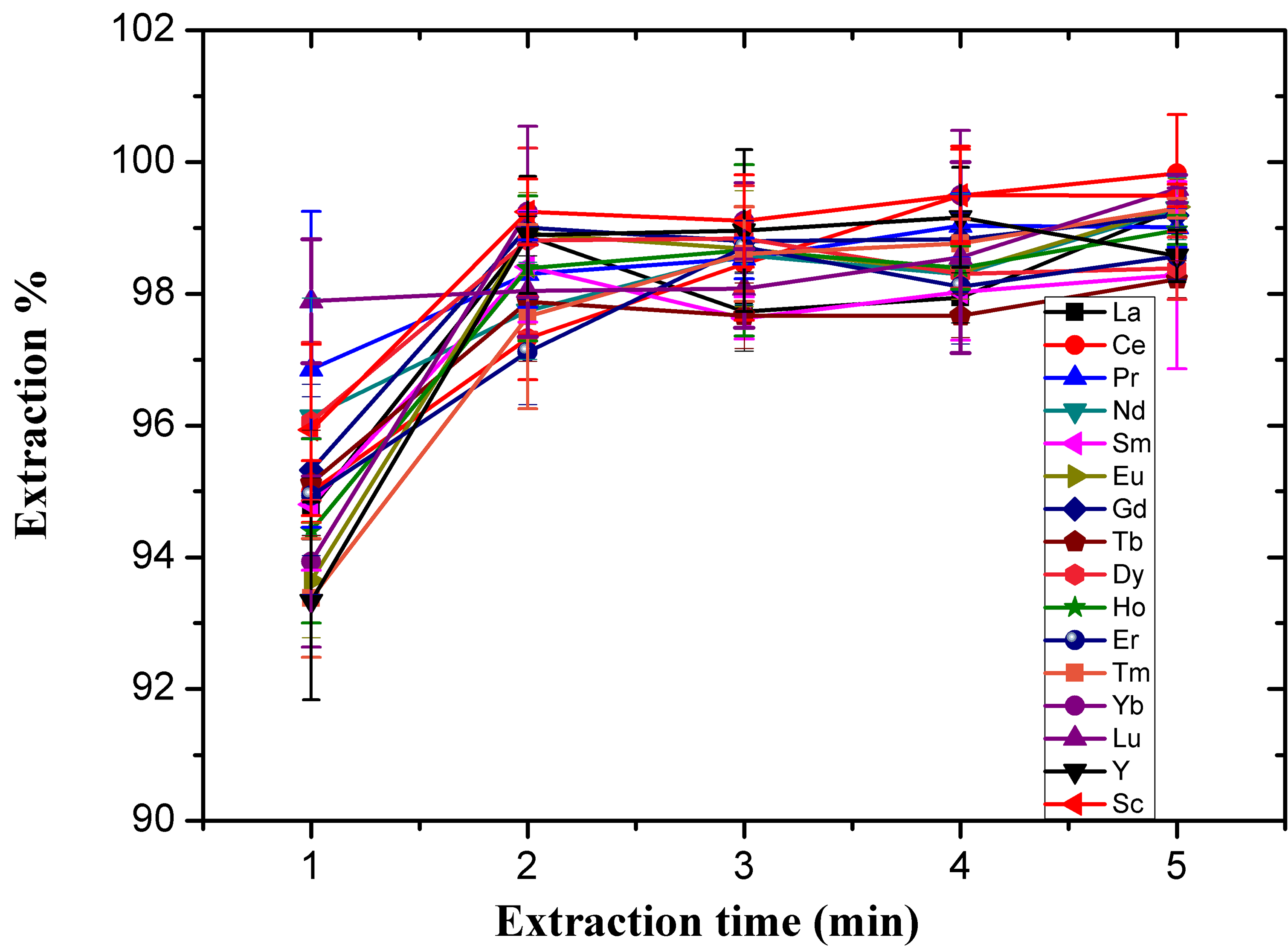

Supplement: S1 Fig — Concentration of each REE: 200 ng mL-1; concentration of HNO3: 3 M; TODGA: 0.025 M; extraction frequency: three times. (TIF) [file pone.0185302.s001.tif]

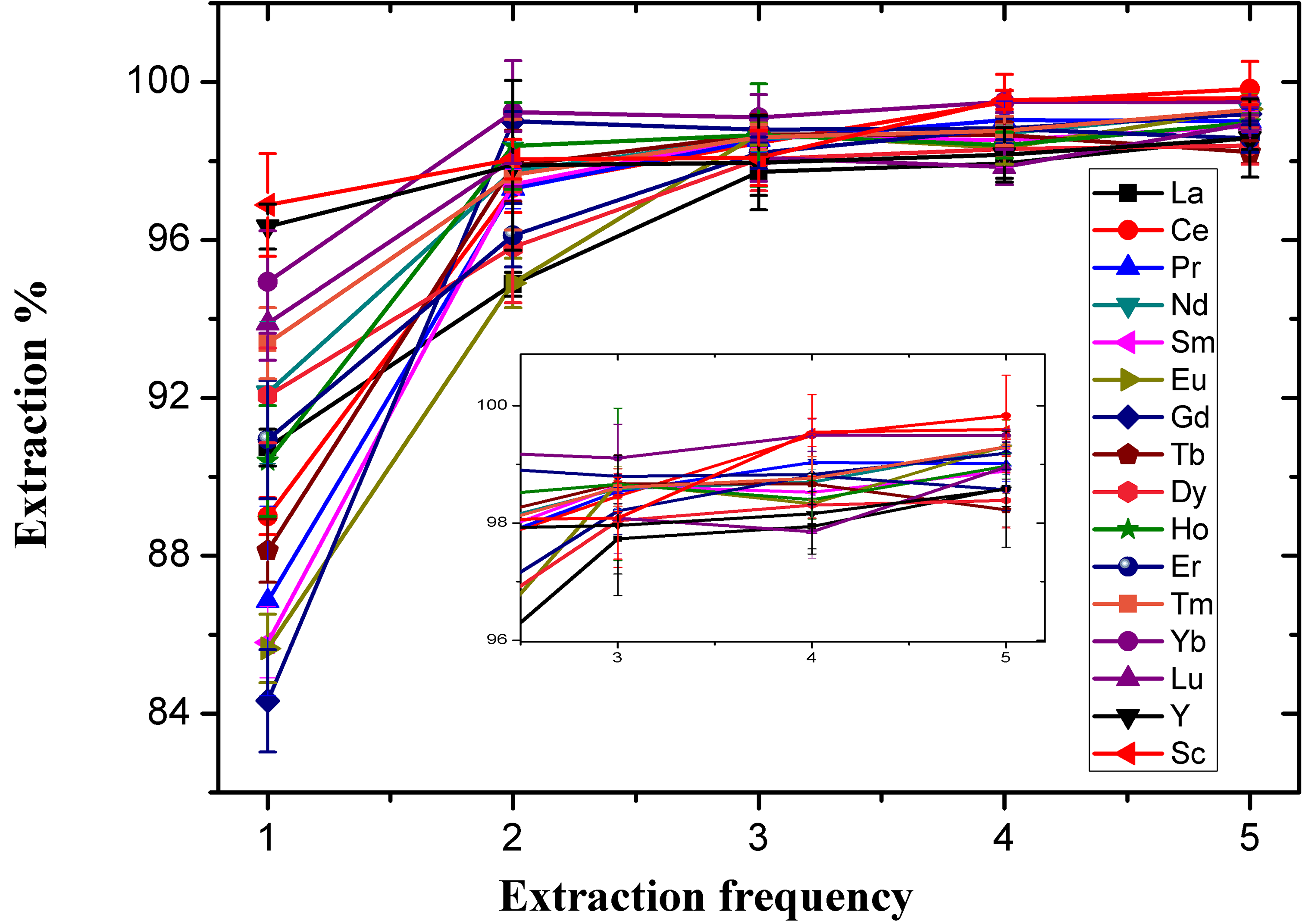

Supplement: S2 Fig — Concentration of each REE: 200 ng mL-1; concentration of HNO3: 3 M; TODGA: 0.025 M; extraction time: 2 min each time. (TIF) [file pone.0185302.s002.tif]

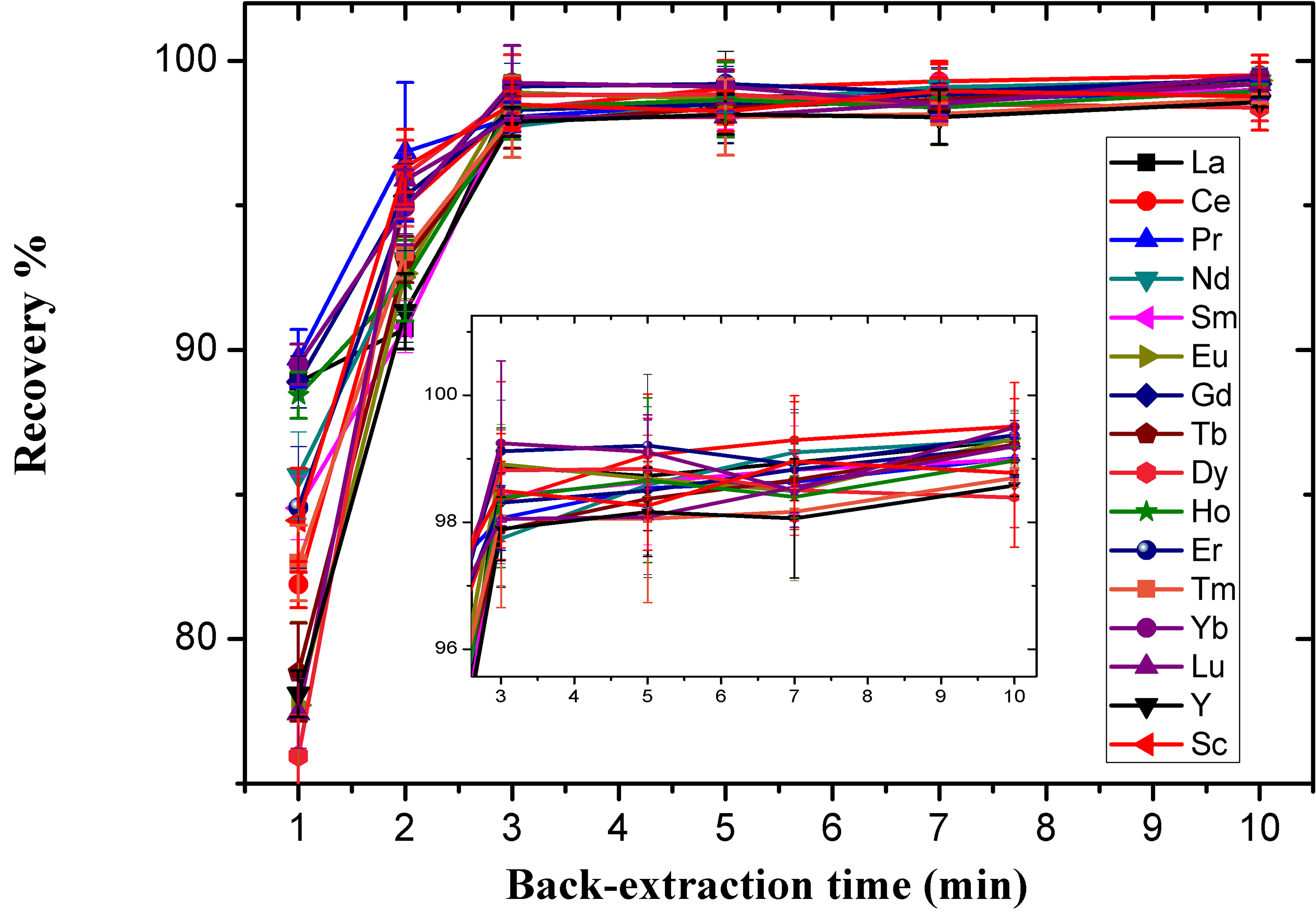

Supplement: S3 Fig — Concentration of each REE: 200 ng mL-1; concentration of HNO3: 3 M; extraction time: 2 min each time; back extraction: 0.03 M EDTA three times. (TIF) [file pone.0185302.s003.tif]

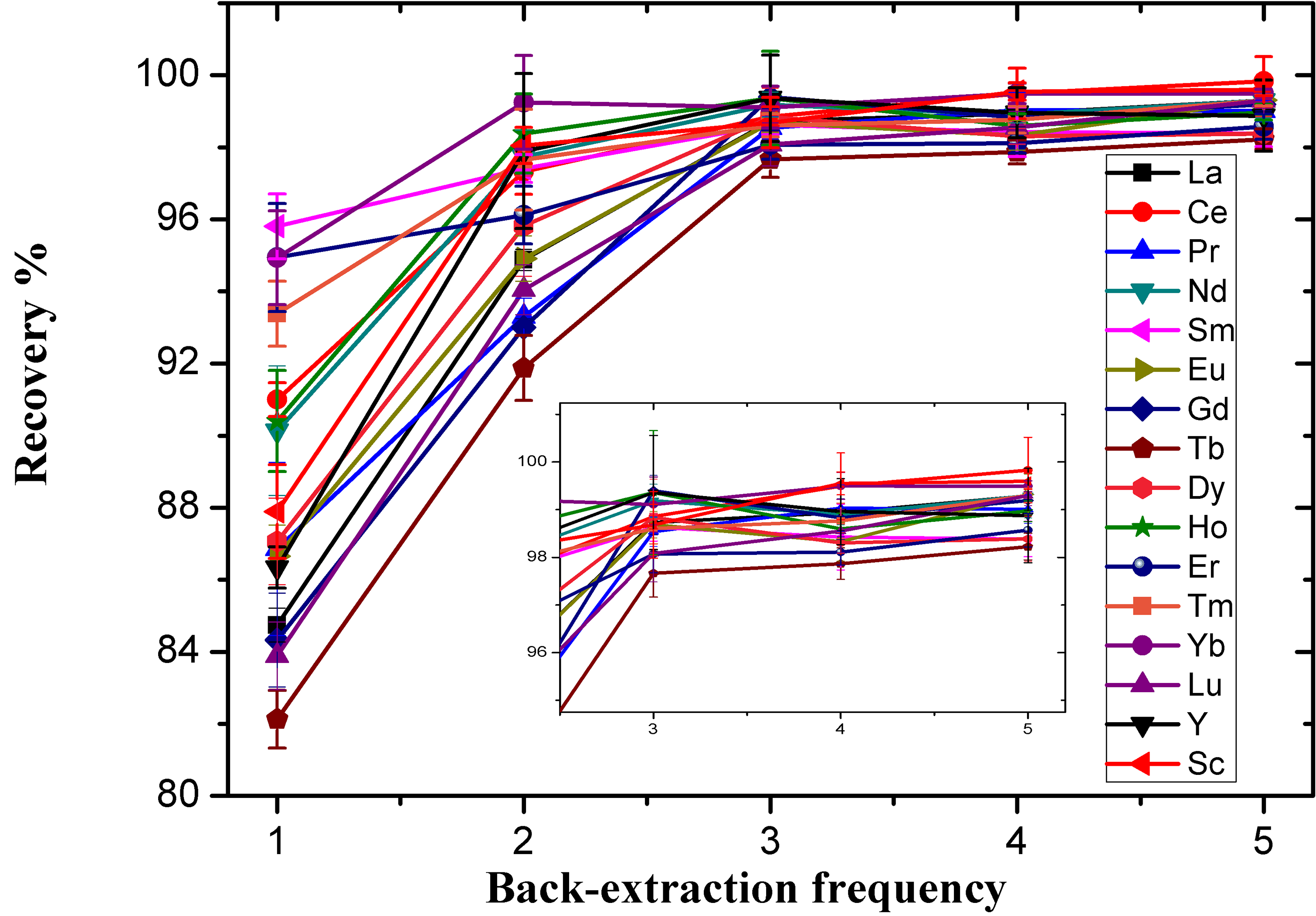

Supplement: S4 Fig — Concentration of each REE: 200 ng mL-1; concentration of HNO3: 3 M; extraction time: 2 min each time; back extraction: 0.03 M EDTA with a stripping time of 3 min. (TIF) [file pone.0185302.s004.tif]
